# Revision of the Cognitive Assessment for Dementia, iPad Version (CADi2)

**DOI:** 10.1371/journal.pone.0109931

**Published:** 2014-10-13

**Authors:** Keiichi Onoda, Shuhei Yamaguchi

**Affiliations:** Department of Neurology, Shimane University, Izumo, Shimane, Japan; McGill University, Canada

## Abstract

Early detection of dementia is crucial because it is the time when intervention is most effective. Therefore, a simple and short test is necessary for primary mass screening in community-based medical facilities. We developed the Cognitive Assessment for Dementia, iPad version (CADi) which consists of 10 simple questions and is self-administered. In this paper we present a revised version which improves the detection of dementia. Two questions of the CADi were replaced in the latest version (CADi2). We examined the validity and reliability of the CADi2 in 27 Alzheimer’s disease patients and age-matched healthy controls. The Alzheimer’s disease patients had lower CADi2 scores and longer total response times to questions compared to the controls. The CADi2 had high sensitivity (0.85−0.96) and specificity (0.81−0.93), and showed significant correlations with existing standard neuropsychological tests. Cronbach’s alpha analysis revealed moderate consistency of the CADi2. These results support the utility of the CADi2 for primary screening for dementia.

## Introduction

Dementia patients have greatly increased as the population has aged [1]. The progression of dementia from Alzheimer’s disease (AD) is irreversible. Recent drug therapies have been most effective when they are initiated early and maintained over time [2]. Therefore, early detection is crucial to implement countermeasures against dementia.

The Mini-Mental State Examination (MMSE) [3] is one of the most widely used screening tools, not only for clinical use but also for use in epidemiological surveys. The MMSE has good sensitivity and specificity for detection of dementia [4]. Several alternative dementia screening methods have been proposed [5]. Some of these are computerized and showed good performance for differentiating between cognitively healthy and impaired elderly [6], [7]. In our county, community-based health checkups are widely prevalent and provide the best opportunity for early detection of dementia. There is therefore a large demand for specialized tests useful for such primary mass screening. Such a screening test requires the following features: 1) easily administered to and understood by elderly persons, 2) should be self-administered without a trained examiner present, 3) brief administration time, and 4) low cost. Existing tests fit these criteria to varying degrees, although they also have significant disadvantages for mass screening purposes.

We developed and proposed a new screening test (Cognitive Assessment for Dementia, iPad version: CADi) that can run on a tablet computer for mass screening [8]. Using the CADi, we performed mass screening for dementia without a trained examiner and in a brief time. The CADi consists of ten separate items, including immediate recognition, long term memory, categorization, subtraction, backward repetition, cube rotation, pyramid rotation, making sequences, and delayed recognition. The CADi score ranges from 0 to 10 correct responses. The sensitivity and specificity of the CADi score for dementia were 0.90 and 0.82, respectively. Additionally, the CADi score significantly correlated with the MMSE score (r = 0.74). The evidence shows that the CADi is useful for dementia screening.

We decided to revise the CADi to improve its discrimination performance. We deleted two items (categorization and pyramid rotation) because of their low contribution to discrimination [8]. We added two new items concerning orientation in time (month, day of week) because people with dementia can experience difficulty with orientation.

The following items make up the revised CADi; items were presented as text on the CADi screen and/or by audio through headphones.

Item 1: Three words (cat, bus, and orange) are presented via audio slowly, one at a time. The list is presented twice. The participant is asked to select the three studied words from a set of six choices (cat, dog, bus, train, apple, and orange). Then the participant is instructed to remember these three words because they will be asked to recall them later.

Item 2: The participant is asked to provide the date of termination of hostilities in World War II. The participant chooses the correct answer from a list of months (July, August, September, and October) and days (6th, 9th, 15th, and 18th).

Item 3: Three digits (5, 1, and 8) are presented via audio slowly, one at a time. The participant is then asked to key in the digits one at a time in reverse order.

Item 4: The participant is asked to choose the present month from six choices provided.

Item 5: The participant is asked to choose the present day of the week from seven choices provided.

Item 6: The participant is asked to choose the answer to the problem “93 minus 7” presented from four choices (84, 85, 86, and 87).

Item 7: Four three-dimensional shapes (two cubes depicted from different viewpoints, a rectangular parallelepiped, and a trapezoid corpus) are presented. The participant is asked to choose the pair of matching objects.

Item 8: Six digits (1, 2, 3, 4, 5, and 6) are presented at random positions on the screen. The participant is asked to touch the digits on the screen from 1 to 6 in sequential order.

Item 9: Three digits (1, 2, and 3) and three Japanese hiragana characters (a, i, and u) are presented at random positions on the screen. The participant is asked to touch the digits and hiragana characters on the screen, alternating between the two in sequential order (1, a, 2, i, 3, u).

Item 10: The participant is again asked to select the three words presented in Question 1 from among the six choices.

Thus, the CADi was reconstructed in this manner and named the CADi2. The aim of the current study is to investigate the validity and reliability of the CADi2 for detection of dementia.

## Methods

### Participants

Patients with mild and moderate Alzheimer’s disease (AD) and age-matched healthy controls (HC) were included in the present study. The AD subjects were recruited from the outpatients of Shimane University Hospital. Practicing neurologists diagnosed all the AD patients based on medical history, a functional assessment, clinical examination, neuroimaging (MRI and SPECT), and neuropsychological tests, including the MMSE and Clinical Dementia Rating (CDR) scale. The neuropsychological tests were administered to each patient individually by an experienced examiner. The AD patients met the criteria for dementia as described in the DSM-IV (Diagnostic and Statistical Manual of Mental Disorders, Fourth Edition) and NINCDS-ADRDA (National Institute of Neurological and Communicative Disorders and Stroke and the Alzheimer's Disease and Related Disorders Association). We recruited HC participants from individuals presenting for routine brains scans as part of a checkup. These individuals underwent neuropsychological testing, MRI, and a clinical examination. The neuropsychological batteries for dementia and control patients consisted of different tests with some exceptions (MMSE, etc.). The inclusion criteria for the HC group were a MMSE score above 26, and we confirmed that these participants did not have memory deficits using the Wechsler Memory Scale concise version (> = 40: normal) [9]. Brain atrophy was independently estimated by visual inspections from two trained doctors (a neurologist and a radiologist), who judged whether observed brain atrophy was consistent with age. Based on this decision, we excluded participants with atrophy from further analysis. The exclusion criteria for both AD and HC were as follows: 1) age above 85 years old because of the difficulty of recruiting cognitively healthy elderly in this age range, 2) decline in vision, 3) hypoacusis, 4) motor deficits, 5) noncompliance with the testing, and 6) existence of other neurological and/or psychiatric history. Twenty-seven patients and the same number of healthy elderly were assigned to the AD and HC groups. The mean ages were 78.1±4.4 and 76.0±3.0 years old for the AD and HC groups. The sex ratios (F/M) were 13/14 for the AD group, and 14/13 for the HC group. Participants received verbal and written descriptions of the study and provided their written informed consent. When some participants were unable to understand the explanation, family members provided consent and signed the document. The medical ethics committee of Shimane University approved the study protocol.

### CADi2 and other neuropsychological tests

The CADi2 was administered to the participants by an examiner who understood the items and who was instructed to help the participants as necessary, with the exception of indicating responses to the items. The examiner performed initial operations such ID input and volume control. After that, the participants were asked to use the CADi2 by touching the screen. All CADi2 instructions and items were presented as text on the tablet screen as well as via audio through headphones. Before the actual items, the participants did a screen touch and two rehearsal items as practice. If a participant complained of not being able to understand a question, the examiner rephrased it and provided encouragement. The CADi2 automatically recorded correct and incorrect responses and the response time for each item. The response time was defined as the interval from when the answer options were available to the end of the selection. The participants could touch the answer options during the voice presentation of the question statement.

All participants underwent some common neuropsychological tests, including the MMSE, the Frontal Assessment Battery (FAB) [10], and a verbal fluency task (VFT; words in the vegetable category) [11]. The Clock Drawing test [12] and Trail Making test [13] were included in the neuropsychological battery for the dementia patients, while the Wechsler memory scale concise version [9] was administered to control participants. Two affective indices (self-rating depression scale: SDS [14] and apathy scale: AS [15]) were administered to both groups.

### Statistical analysis

We used t-tests and kai^2^ tests to compare the demographic and neuropsychological data. Because we found a significant education difference between the groups, ANCOVAs were used to evaluate the neuropsychological indices. We compared the CADi2 scores and the total response times (TRT) between the AD and HC groups to assess discriminability and performed Receiver Operating Characteristic (ROC) analyses. The resultant sum of both the normalized score and TRT (z-scores) was entered into the ROC analysis. To further improve discrimination accuracy, we applied the support vector machine (SVM) to the CADi2 data. The SVM is a machine learning approach that finds a best classifier based on a maximal margin of separation between the two groups [16]. The SVM lets us know the extent to which variables are relevant for discriminating between groups. The AD and HC groups were assigned to +1 and −1, respectively. We used CADi2 score and TRT as features and selected the leave-one-out cross-validation method to estimate the generalization ability of our classifier. A permutation test (1000 iterations) was performed in order to verify the significance of the classifier results. The contribution ratios of score and TRT were then estimated for all participants. The individual weighted vector in a feature space was calculated as a sum of products with the contribution ratios and observed values. We also performed ROC analysis for the resultant value based on SVM-weighted CADi2 score and TRT. We also performed Spearman’s correlation analyses for the CADi2 and performance on the other neuropsychological tests to assess external validity. The statistical criteria were corrected using the Bonferroni method (0.05/32 based on the number of the correlation tests). Finally, to estimate internal consistency, we calculated Cronbach’s alpha for all the items as a whole and for the remaining items after one was removed.

## Results

The demographic and neuropsychological characteristics of the AD and HC groups are presented in Table 1. There were no significant differences in age and sex between the two groups. The AD group had significantly fewer years of education than the HC group. Scores on the cognitive tests were lower for the AD group compared with the HC group (all *ps*<0.001). These differences were significant even after controlling for the effects of age, sex, and education (see Table S1). In addition, we divided the AD group into mild (CDR≦1) and moderate (CDR = 2) AD groups and compared neuropsychological test scores across the three groups. We found that compared with the HC group, the CADi indices for both the mild and moderate AD groups indicated significant impairment (see Table S2). There were no significant affective score differences between the groups.

**Table 1:** Demographic and neuropsychological characteristics of Alzheimer’s disease and healthy control groups.

	Alzheimer’s disease	Healthy control	p-value
Age	78.1±4.4	76.0±3.0	n.s.
Sex (M/F)	14/13	13/14	n.s.
Education (ys)	9.5±2.1	11.6±2.9	0.007
CDR	1.3±0.5	−	
MMSE	17.9±3.9	28.8±1.2	<0.001
FAB	10.3±3.7	16.0±1.2	<0.001
VFT	7.9±3.4	14.3±3.2	<0.001
CDT	10.7±3.3	−	
TMT	99.9±51.2	−	
WMS	−	46.7±5.1	
SDS	33.5±9.5	35.9±6.3	n.s.
AS	12.5±7.5	11.3±5.6	n.s.

CDR: Clinical Dementia Rating, MMSE: Mini-Mental State Examination, FAB: Frontal Assessment Battery, VFT: Word Fluency Task, CDT: clock drawing test, TMT: trail making test (A), WMS: Wechsler Memory Scale concise version. SDS: self-rating depression scale, AS: apathy scale, –: unavailable.

**Figure 1:**
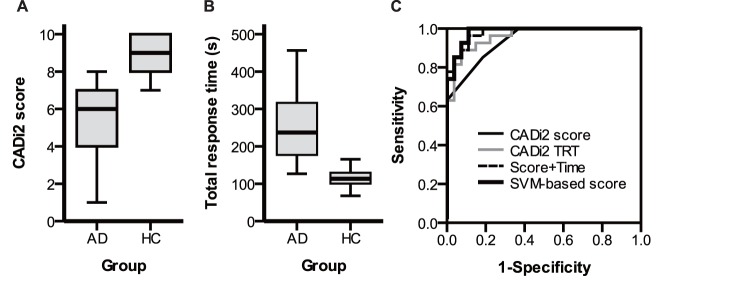
Comparisons of the CADi2 score (A) and total response time (B) for AD and HC groups. C: receiver-operating characteristic curve for each index. AD: Alzheimer’s disease, HC: healthy control, TRT: total response time, SVM: support-vector machine.

The correct response ratio and response time for each question and the total score on the CADi2 are summarized in Table 2. Similar to the other neuropsychological tests, the CADi2 score for the AD group was significantly lower than the HC group (*t*(52) = 7.7, *p*<0.001, *d* = 2.1, Figure 1A). The TRT was significantly longer for the AD group than the HC group (*t*(52) = 7.3, *p*<0.001, *d* = 1.98 Figure 1B). Even after controlling for age, gender, and education years using ANCOVA, the group effects on CADi scores and TRT were significant (*Fs*(1, 44)>31.6, *p*s <0.001). We created two indices to improve the accuracy of discrimination between the two groups. One was the sum of the normalized score and the TRT, and the other was the SVM-based weighted vector. The performances of the SVM linear classifier in the leave-one-out cross-validation method were 0.89 for both sensitivity and specificity. The permutation test revealed the classifier’s strong significance for the discrimination (*p*<0.001). The SVM-weighted vector of the individual was finally calculated as follows: −0.9987 * CADi2 Score +0.0505 * TRT (sec). We performed the ROC analyses for these four indices (Figure 1C; Table 3). At a score of 7 or less, the sensitivity and specificity were 0.85 and 0.81, respectively. Similarly, those of the TRT were 0.89 and 0.93 (>157s). Integrating the score and the TRT improved the ROC indices. The SVM-based weighted score showed a good performance as same as the sum of the normalized score and the TRT.

**Table 2:** Correct response rate, response time, and Cronbach’s alpha of CADi2.

	Correct response			Response time			Cronbach’s alpha
	AD	HC	p-value	AD	HC	p-value	
1. Immediate recognition	0.82	1.00	0.38	20.7	10.8	<0.001	0.74
2. Long term memory	0.78	0.89	0.273	30.2	12.2	<0.001	0.74
3. Digits backward	0.37	0.85	<0.001	23.7	11.7	<0.001	0.73
4. Orientation (month)	0.52	1.00	<0.001	17.5	7.0	<0.001	0.73
5. Orientation (day of week)	0.52	0.93	0.001	21.4	6.8	<0.001	0.72
6. Calculation	0.59	0.81	0.074	39.4	13.3	<0.001	0.75
7. Cube rotation	0.89	1.00	0.075	17.8	10.3	<0.001	0.76
8. Sequence making A	0.78	0.93	0.125	27.9	13.4	0.003	0.75
9. Sequence making B	0.07	0.52	<0.001	25.4	17.6	0.023	0.73
10. Delayed recognition	0.11	0.89	<0.001	32.4	15.1	<0.001	0.70
Total	5.48	8.81	<0.001	256.3	117.8	<0.001	0.75

AD: Alzheimer’s disease, HC: healthy control.

**Table 3:** Results of ROC analysis.

	SNS	SPC	ACC	AUC
Score	0.85	0.81	0.83	0.94
TRT	0.89	0.93	0.91	0.96
Score+TRT	0.96	0.89	0.93	0.98
SVM weighted vector	0.93	0.93	0.93	0.98

SNS: sensitivity, SPC: specificity, ACC: accuracy, AUC: area under the curve, TRT: total response time, SVM: support vector machine.

The correlation analyses between the CADi2 indices and neuropsychological tests are summarized in Table 4. The CADi2 indices had significant high correlations with the other cognitive scores (|*rs*|>0.71 *ps*<0.001). Similar to the ROC analyses, the correlation coefficients were higher for the integrated indices than the single ones.

**Table 4:** Correlation coefficient between CADi2 and neuropsychological tests.

	Score	TRT	Score +TRT	SVM-based index
MMSE	0.79*	−0.78*	0.84*	−0.84*
FAB	0.76*	−0.83*	0.85*	−0.86*
VFT	0.67*	−0.58*	0.67*	−0.65*
CDT	0.27	−0.21	0.26	−0.25
TMT	−0.33	0.47†	–0.46†	0.47†
WMS	0.30	−0.50†	0.46†	−0.55†
SDS	0.14	−0.17	0.19	−0.19
AS	−0.04	0.07	–0.07	0.06

TRT: total response time, SVM: support vector machine, MMSE: Mini-Mental State Examination, FAB: Frontal Assessment Battery, VFT: verbal Fluency Task, CDT: clock drawing test (Alzheimer’s Disease group only), TMT: trail making test (A; Alzheimer’s Disease group only), WMS: Wechsler Memory Scale concise version (Healthy Control group only). SDS: self-rating depression scale, AS: apathy scale, *: corrected p<0.05, †: uncorrected p<0.05.

To examine the internal consistency, Cronbach’s alpha was calculated for the whole instrument and calculated after each item was deleted from the total item pool (right-most column of Table 2). The alpha for each item fell into the range of 0.70–0.76, and there was no remarkable outlier.

## Discussion

This study examined the validity and reliability of the CADi2 for screening for dementia. The CADi2 showed high sensitivity and specificity for distinguishing between the Alzheimer’s dementia and the healthy control groups, and significant correlations with existing neuropsychological tests. Also, the Cronbach’s alphas fell within the adequate range. These results indicate that the CADi2 has high validity and reliability for detection of dementia.

For this revision of the CADi, two items (categorization and pyramid rotation) were removed because of the low distributions [8] for discrimination between participants with dementia and healthy elderly participants, and two new items of orientation in time were added. An orientation deficit is one of the symptoms which emerges early in the progression of Alzheimer’s disease. The MMSE suitably includes orientation about the year, month, day and the day of week [3]. In our data, almost all healthy elderly individuals could correctly answer the questions about the month and day of the week, whereas half of the Alzheimer’s disease patients made mistakes (see Table 2). Additionally, the CADi2 has moderate Cronbach’s alphas, and there was no remarkable high value from deleting each item. This shows that the CADi2 has good internal consistency, and the two new items are suitable for dementia screening.

The CADi2 yielded significantly lower scores for the patients with Alzheimer’s dementia than the healthy control group. Also, the total response time on the CADi2 was significantly longer for the patients with Alzheimer’s dementia. The ROC analysis revealed that the CADi2 score and the total response time had high sensitivity and specificity for discrimination between the patients with Alzheimer’s dementia and the healthy controls. This suggests that the CADi2 has adequate construct validity. Our previous study reported that the sensitivity and specificity of the original CADi score were 0.96 and 0.77, respectively. Compared to the performance of the original CADi, the specificity was improved but the sensitivity was decreased. The decreased sensitivity might have been affected by limiting the Alzheimer’s dementia group not to severe but to mild and moderate Alzheimer’s disease, but even so it was high enough. On the other hand, the increased specificity is assumed to be due to the conversion to easier questions (orientation) for the elderly with normal cognition. Also, the CADi2 score showed lower sensitivity and specificity than the total response time. Because of the space limitations of the tablet monitor, the number of response alternatives was small. The probability that a participant accidently pressed the correct answer may increase on the CADi2. Therefore, the time required to answer questions should be also considered for screening using the CADi2. A decline of processing speed in dementia has been shown repeatedly [17], [18]. In the current study, integrating the score and the total response time improved the discrimination performance. Especially, the weighted vector based on SVM pattern recognition showed good sensitivity and specificity. This result suggests that integrating scores with adequate weightings might lead to better screening for dementia.

The CADi2 indices were significantly correlated with MMSE, FAB, and VFT scores. The correlation coefficients were higher than those observed for the previous version [8]. This result indicates that the CADi2 has good concurrent validity. The MMSE is also valuable in the detection of dementia and has been a standard for estimating general cognitive abilities. However, the MMSE cannot be used alone as a diagnostic tool because the outcomes are affected by age, education, and cultural background [19]. The same concerns could be applied to the CADi2. In the present study, Alzheimer’s disease patients were less educated than age-matched healthy elderly, although the test successfully differentiated between the groups when education level was controlled. Additionally, the CADi2 is optimized for administration to Japanese elderly due to inclusion of item 2 (the end date of World War II is common knowledge in Japan). Thus, this tool should be used only for primary screening for dementia rather than for deriving an ultimate diagnosis.

Another important point concerns mild cognitive impairment (MCI). The early detection of dementia has at least two aspects. One concerns the detection of individuals with MCI who have memory deficits but no significant functional impairment. Another issue is the detection of potential dementia patients who require medication treatment as early as possible. We have operated the previous version of the CADi in community-based medical checkups (n = 2000 per year) and followed people with low CADi scores. Empirically, the cost of making a definite diagnosis of MCI is much higher, given that the discrimination accuracy of the CADi is lower for MCI compared to dementia. A similar result has been reported for another computerized test [20]. Optimization for detection of early AD might be more cost-effective in mass screening contexts such as health checkups, as compared to screening for MCI. Therefore, we focused on improving the accuracy of the CADi2 for AD detection, and verified that the CADi2 indeed shows good performance for AD. Further work should examine the potential utility of the CADi2 for detection of MCI and non-AD forms of dementia.

We have proposed that the CADi2 is a useful screening test that can be administered without a trained tester; however, this does not mean that the CADi2 can be completely self-administered by anyone. Persons with decreased cognitive functions might encounter difficulties in understanding the instructions and questions. In these situations, self-administration without a tester is not feasible, and the subjects may need the assistance of by a tester. Even then however, CADi2 testers are not necessarily required to be professionals, but only need to understand the content on ahead.

In summary, we developed the CADi2 as a mass-screening test for dementia as part of community-based medical checkups. As we already mentioned above, the CADi2 might have some limitations, including effects of cultural background and education level. However, we believe that the CADi2 is a useful instrument for mass-screening for dementia because it does not require a professional examiner and the administration cost is low. The CADi2 has already been released in the App Store (https://itunes.apple.com/us/app/cadi2/id808586504, currently only a Japanese version).

## Supporting Information

Table S1 **F-values of ANCOVAs for cognitive indices.**
(DOCX)

Table S2 **Demographic and neuropsychological comparisons among 3 groups.**
(DOCX)

Data S1 **Raw data.**
(XLSX)
