# Supplementary material for: Revision of the Cognitive Assessment for Dementia, iPad Version (CADi2)
Source: PLoS One. 2014 Oct 13;9(10):e109931. doi: 10.1371/journal.pone.0109931 (PMC4195614; doi:10.1371/journal.pone.0109931)
Supplement: Table S1 — F-values of ANCOVAs for cognitive indices. (DOCX) [file pone.0109931.s001.docx]

Table S1. F-values of ANCOVAs for cognitive indices.

|  | Age | Sex | Education | Group |
| --- | --- | --- | --- | --- |
| CADi2 score | 0.0 | 0.0 | 1.7 | 35.6* |
| CADi2 TRT | 1.9 | 2.2 | 4.0 | 31.6* |
| MMSE | 1.3 | 1.7 | 1.0 | 136.0* |
| FAB | 0.0 | 0.0 | 2.5 | 38.2* |
| VFT | 0.0 | 1.8 | 0.8 | 38.6* |

TRT: total response time, MMSE: Mini-Mental State Examination, FAB: Frontal Assessment Battery, VFT: Word Fluency Task, *: p<0.05.
