# Supplementary material for: Revision of the Cognitive Assessment for Dementia, iPad Version (CADi2)
Source: PLoS One. 2014 Oct 13;9(10):e109931. doi: 10.1371/journal.pone.0109931 (PMC4195614; doi:10.1371/journal.pone.0109931)
Supplement: Table S2 — Demographic and neuropsychological comparisons among 3 groups. (DOCX) [file pone.0109931.s002.docx]

Table S2. Demographic and neuropsychological comparisons among 3 groups.

|  | HC | Mild AD | Moderate AD | Statistic |
| --- | --- | --- | --- | --- |
| n | 27 | 18 | 9 |  |
| Age | 76.7 ± 3.4 | 78.2 ± 3.9 | 78.0 ± 5.6 | n.s. |
| Sex (M/F) | 13/14 | 9/9 | 5/4 | n.s. |
| Education (y) | 11.6 ± 2.9 | 9.4 ± 2.4 | 9.5 ± 1.2 | Mild AD < HC |
| CDR | − | ≦1 | 2 |  |
| MMSE | 28.9 ± 1.2 | 18.8 ± 2.7 | 15.9 ± 5.2 | Moderate AD < Mild AD < HC |
| FAB | 16.1 ± 1.3 | 11.3 ± 2.6 | 8.1 ± 4.6 | Mild and Moderate AD < HC |
| VFT | 14.6 ± 3.4 | 8.9 ± 3.0 | 5.8 ± 3.3 | Moderate AD < Mild AD < HC |
| CDT | − | 10.9 ± 3.4 | 9.7 ± 4.0 | n.s. |
| TMT | − | 105.0 ± 57.3 | 87.2 ± 31.7 | n.s. |
| CADi2 score | 8.8 ± 1.1 | 6.1 ± 1.6 | 4.3 ± 2.1 | Mild and Moderate AD < HC |
| CADi2 TRT | 117.8 ± 13.3 | 241.4 ± 16.3 | 286.0 ± 23.0 | Mild and Moderate AD < HC |
| SDS | 35.9 ± 6.3 | 32.9 ± 8.8 | 34.7 ± 11.9 | n.s. |
| AS | 11.3 ± 5.6 | 11.4 ± 7.2 | 15.1 ± 8.1 | n.s. |

CDR: Clinical Dementia Rating, MMSE: Mini-Mental State Examination, FAB: Frontal Assessment Battery, VFT: Word Fluency Task, CDT: clock drawing test, TMT: trail making test (A), WMS: Wechsler Memory Scale concise version. SDS: self-rating depression scale, AS: apathy scale, −: unavailable.
